# Effects of benzene, quercetin, and their combination on porcine ovarian cell proliferation, apoptosis, and hormone release

**DOI:** 10.5194/aab-62-345-2019

**Published:** 2019-06-14

**Authors:** Adam Tarko, Aneta Štochmal'ová, Katarína Jedličková, Sandra Hrabovszká, Adriana Vachanová, Abdel Halim Harrath, Saleh Alwasel, Abdulkarem Alrezaki, Jan Kotwica, Andrej Baláži, Alexander V. Sirotkin

**Affiliations:** 1 Department of Zoology and Anthropology, Constantine the Philosopher University, Tr. A. Hlinku 1, 949 74 Nitra, Slovakia; 2 Institute for Genetics and Reproduction of Farm Animals, Animal Production Research Centre Nitra, Hlohovecka 2, 951 41 Lužianky, Slovakia; 3 Dept. of Zoology, College of Science, King Saud University, Riyadh, Saudi Arabia; 4 Institute of Animal Reproduction and Food Research, Polish Academy of Sciences, Olsztyn, Poland

## Abstract

We hypothesized that the environmental contaminant benzene and the plant antioxidant quercetin may affect ovarian cell functions and that quercetin could offer protection against the adverse effects of benzene. This study aimed to examine the action of benzene, quercetin, and their combination on porcine ovarian granulosa cell functions. We elucidated the effects of benzene (20 µg mL${}^{-1}$), quercetin (at the doses 0, 1, 10, 100 µg mL${}^{-1}$), and their combination on ovarian granulosa cell functions (proliferation, apoptosis, and hormone release) in vitro using immunocytochemistry and enzyme immunoassay respectively. Benzene alone stimulated proliferation, apoptosis, and oxytocin release and inhibited progesterone and prostaglandin F release. Quercetin alone inhibited proliferation, apoptosis, and stimulated oxytocin release but did not affect progesterone and prostaglandin F release. When used in combination with benzene, quercetin promoted the inhibitory effect of benzene on progesterone release. Overall, these data suggest that benzene and quercetin have direct stimulatory and inhibitory effects, respectively, on basic ovarian functions. Moreover, no protective action of quercetin against the effects of benzene was found. Rather, it was found to enhance the effect of benzene on progesterone release. Therefore, quercetin cannot be considered for preventing or mitigating the effects of benzene on reproductive processes.

## 1 Introduction

Benzene is a common industrial chemical, a component of gasoline, and a constituent of engine emissions and tobacco smoke (Kalf, 1987). It is a known human carcinogen and numerous case reports and epidemiological studies have provided evidence of a causal relationship between exposure of females to benzene and abnormal menstrual cycles, severe bleeding, convulsions, and a higher rate of aborted pregnancies. The influence of benzene on the female reproductive system, in particular on fertility (Mukhametova and Vozovaya, 1972; Vara and Kinnunen, 1946) and the menstrual cycle (Michon, 1965), has been previously examined, but these studies did not generate definitive conclusions. The mechanisms underlying the action of benzene on the female reproductive system remain unclear. Oil-related contaminants can affect the female reproductive system by direct action on ovarian cells via ovarian functions regulated by upstream hypothalamo-hypophysial system (Sirotkin and Harrath, 2014). The target of benzene action remains to be established. If the direct action of benzene on the ovary could be detected, its mechanisms would require further examination because ovarian functions are regulated by ovarian cell proliferation, apoptosis, and secretory activity (Sirotkin, 2014). To our knowledge, there have been no studies exploring the mechanisms underlying the effects of benzene on the female reproductive system. Therefore, there is an urgent need to establish strategies for neutralizing the negative effects of benzene on the female reproductive system. Some plant molecules can protect cells from the negative effects of environmental stressors. For example, the flavonoid quercetin, which is present in fruits, vegetables, and beverages, can improve animal reproductive function (Dhawan et al., 2002) via anticancer (Aalinkeel et al., 2008; Jeong, 2008; Jeong et al., 2009; Warren, 2009), antihypertensive (Mackraj et al., 2008), anti-inflammatory (Kumazawa et al., 2006), and antimicrobial (Davis et al., 2008) effects. It may do so, for example, by suppressing tumour cell proliferation (Jeong, 2008; Warren, 2009). The action of quercetin on healthy ovarian cells has been demonstrated previously on rabbit (Leśniak-Walentyn et al., 2013), mouse (Shu et al., 2011) and bovine (Tarko et al., 2018) ovaries; however, the action of quercetin on porcine reproductive processes and the ability of quercetin to protect these processes against environmental contaminants, including benzene, have not yet been studied.

The main aim of this study was to evaluate the effect of benzene, quercetin, and their combination on basic ovarian cell functions (proliferation, apoptosis, hormone release) and to examine the possible protective effect of quercetin against benzene action.

## 2 Material and methods

### 2.1 Preparation, processing, and culture of ovarian granulosa cells

We obtained 12 ovaries from 3- to 5-month-old Landrace breed specimens at the follicular stage of the estrous cycle from the slaughterhouses of the National Agricultural Food Centre in Nitra and in Stará Myjava.

The ovarian granulosa cells were prepared according to Tarko et al. (2017). After 96 h of culture, when the cells created a monolayer of 75 %, the medium was replaced with a fresh one that was supplemented with one of the following treatment conditions: (1) control groups without treatment of quercetin (Q) (for research use only, sc-206089A, Santa Cruz Biotechnology) extract (AppliChem GmbH, Germany; 0 µg mL${}^{-1}$ of quercetin), (2) groups treated with Q extract (1, 10, and 100 µg mL${}^{-1}$), (3) a control group treated with benzene (20 and 0 µg mL${}^{-1}$ of Q) (Reag. Ph. Eur., 99 % for analysis, ACS, ISO, AppliChem ITW Reagents Germany), and (4) groups treated with benzene (20 µg mL${}^{-1}$) (99 % Reag. Ph. Eur. for analysis, ACS, ISO, AppliChem ITW Reagents Germany) and quercetin extract (1, 10, and 100 µg mL${}^{-1}$). After incubation for 48 h, the medium was removed from the culture plates using a syringe and stored at -70 ${}^{\circ }$C until EIA. The granulosa cells on the chambered slides were ICC treated.

### 2.2 Immunocytochemistry (ICC)

Proliferation (PCNA) and apoptosis (BAX) markers were detected via immunocytochemistry using the method described by Osborn and Isenberg (1994) and Tarko et al. (2017). The presence of markers of proliferation and apoptosis was detected by immunocytochemistry (Osborn and Isenberg, 1994). After washing and fixation, the cells were incubated in blocking solution (1 % goat serum (Santa Cruz Biotechnology Inc., Dallas, TX, USA) in PBS) at room temperature for 1 h to block non-specific binding of the antiserum. The cells were incubated at the presence of monoclonal antibodies against either PCNA (dilution 1:500 in PBS; cat. no. sc-25280, Santa Cruz Biotechnology Inc., Dallas, TX, USA) or BAX (dilution 1:500 in PBS; cat. no. sc-23959, Santa Cruz Biotechnology Inc., Dallas, TX, USA). After 1 h of incubation at room temperature, cells were incubated with a secondary swine anti-mouse IgG (dilution 1:1000; cat. no. sc–2031, Santa Cruz Biotechnology Inc., Dallas, TX, USA) labelled with horseradish peroxidase (HRP; Servac, Prague, Czech Republic) for 1 h. Positive signals were visualized by staining with 3,3'-diaminobenzidine (DAB) (K3468) substrate (Roche Diagnostics GmbH, Mannheim, Germany) for 1 h. After DAB staining, the cells on the chamber slides were washed in PBS, and then covered with a drop of Glycergel Mounting Medium (DAKO, Glostrup, Denmark), and then a cover slip was attached to a microslide. The presence and localization of PCNA and BAX positive cells was proved on the basis of HRP–DAB (brown staining). Cells processed without the primary antibody were used as a negative control. A ratio of HRP–DAB-stained cells to the total cell number was calculated.

## 3 Enzyme immunoassay (EIA)

Concentrations of progesterone, oxytocin, and prostaglandin F were determined in 25–100 µL samples of the incubation medium by enzyme immunoassay (EIA)/radio immunoassay (RIA), as described previously by Prakash et al. (1987).

The EIA for P, OT, and PGF was based on the paper of and performed in accordance to Prakash et al. (1987) with our slight modifications (Kotwica et al., 1993, 1994). The cross-reactivity of the antisera used against OT and P4 were previously reported by Kotwica et al. (1993, 1994), and those against plasma 13,14-dihydro-15-keto alpha (PGFM) were reported by Homanics and Silvia (1988). The range of the standard curve, the intra- and inter-assay coefficients of variation, and the relationship between the added and measured hormone concentrations (n=4), expressed as the coefficient of regression, were as follows: for P4, 0.37–25 ng mLl${}^{-1}$, 8.7 %, 10.2 %, and r=0.96; for OT, 3.9—1000 pg mLl${}^{-1}$, 9.8 %, 10.8 %, and r=0.94; for PGFM, 62.5–2000 pg mLl${}^{-1}$, 8.2 %, 11.9 %, and r=0.98, respectively. All the assays were previously validated for use by in serial dilution tests. All assays were previously validated for use by serial dilution in samples of blood plasma (PBSs) or culture media (samples collected during in vitro experiments).

## 4 Statistical analysis

We performed three independent repetitions for ICC experiments and four EIA repetitions. In the independent experiments, each experimental group was represented by four culture wells for enzyme immunoassays or one chamber slide well for immunocytochemistry according Tarko et al. (2017) using a two-way ANOVA followed by Dunnett's test. In this paper, only the comparison between the following selected groups has been presented: (1) the differences between cells without and with quercetin extract addition at three different levels without benzene, (2) the differences between cells with or without benzene, (3) the differences between cells without and with three different concentrations of quercetin extract with the simultaneous addition of benzene. Values are presented as mean ±SD. Differences between control and experimental groups were considered significant at P<0.05.

## 5 Results

### 5.1 Proliferation

**Figure 1:**
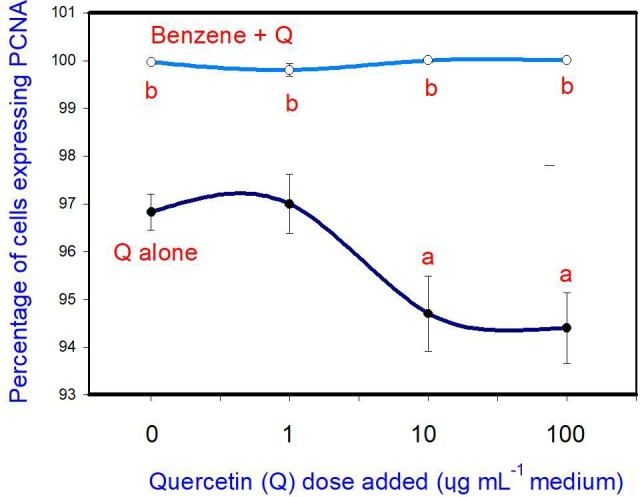
Effects of benzene, quercetin extract, and their combination on cell proliferation in cultured porcine ovarian granulosa cells. Parts marked “a” show the effect of quercetin extract (significant difference (P<0.05) between cells cultured without and with quercetin addition at three different levels without benzene), b the effect of benzene (significant difference, P<0.05) between cells cultured with or without benzene for each quercetin dose separately), and c the effect of quercetin extract with simultaneous benzene presence (significant difference (P<0.05) between a cell cultured with and without quercetin addition at three different levels with simultaneous benzene addition); effect of benzene alone (20 µg mL${}^{-1}$) is displayed for quercetin dose at 0 µg mL${}^{-1}$.

The administration of 20 µg mL${}^{-1}$ of benzene (B) stimulated cell proliferation. Quercetin (Q) inhibited cell proliferation at a concentration of 10 and 100 µg mL${}^{-1}$, but not at 1 µg mL${}^{-1}$. Moreover, when administered in conjunction with B, Q did not significantly modify the stimulatory effect of B on cell proliferation at any dose (Fig. 1).

### 5.2 Apoptosis

**Figure 2:**
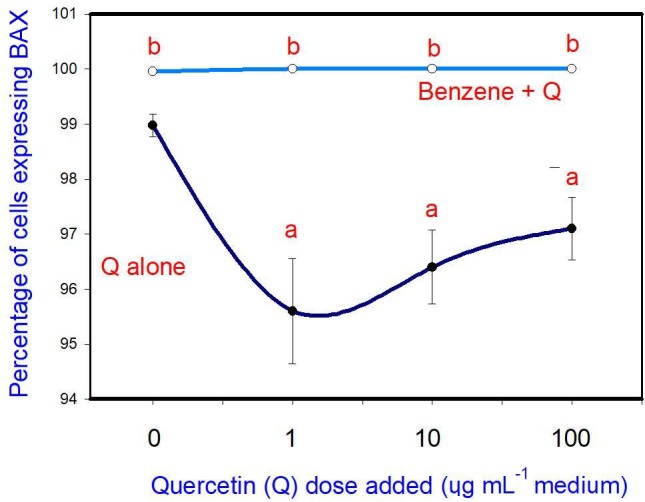
Effects of benzene, quercetin extract, and their combination on apoptosis in cultured porcine ovarian granulosa cells. Parts marked “a” show the effect of quercetin extract (significant difference (P<0.05) between cells cultured without and with quercetin addition at three different levels without benzene), b the effect of benzene (significant difference (P<0.05) between cells cultured with or without benzene for each quercetin dose separately), and c the effect of quercetin extract at simultaneous benzene presence (significant difference (P<0.05) between a cell cultured with and without quercetin addition at three different levels with simultaneous benzene addition); effect of benzene alone (20 µg mL${}^{-1}$) is displayed for quercetin dose at 0 µg mL${}^{-1}$.

The administration of 20 µg mL${}^{-1}$ of B stimulated cell apoptosis. Q inhibited apoptosis at a concentration of 10 and 100 µg mL${}^{-1}$, but not at 1 and 10 µg mL${}^{-1}$. Moreover, when administered in conjunction with B, Q did not significantly modify the stimulatory effect of B on apoptosis at any dose (Fig. 2).

### 5.3 Progesterone

**Figure 3:**
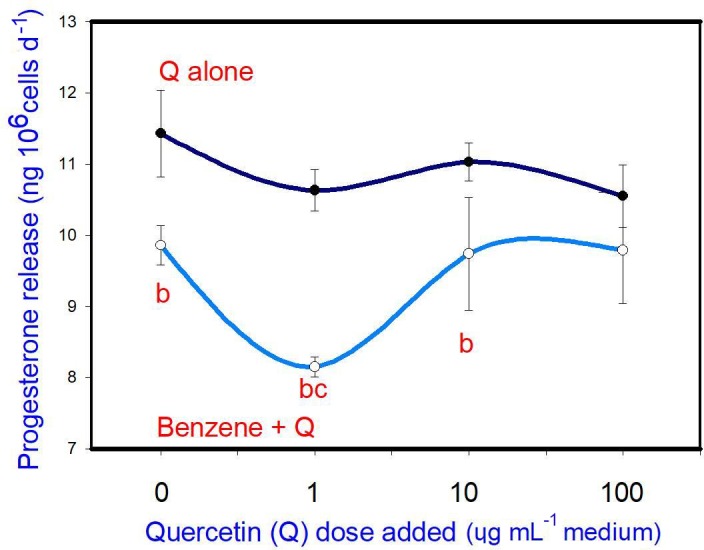
Effects of benzene, quercetin extract, and their combination on the release of progesterone in cultured porcine ovarian granulosa cells. Parts marked “a” show the effect of quercetin extract (significant difference (P<0.05) between cells cultured without and with quercetin addition at three different levels without benzene), b the effect of benzene (significant difference (P<0.05) between cells cultured with or without benzene for each quercetin dose separately), c the effect of quercetin extract at simultaneous benzene presence (significant difference (P<0.05) between a cell cultured with and without quercetin addition at three different levels with simultaneous benzene addition); effect of benzene alone (20 µg mL${}^{-1}$) is displayed for quercetin dose at 0 µg mL${}^{-1}$.

B (20 µg mL${}^{-1}$) inhibited progesterone release. Q, when added alone, did not affect progesterone release at any dose. When administered in conjunction with B, Q promoted the inhibitory effect of B (at 1 µg mL${}^{-1}$, but not at 10 and 100 µg mL${}^{-1}$) on progesterone release (Fig. 3).

### 5.4 Oxytocin

**Figure 4:**
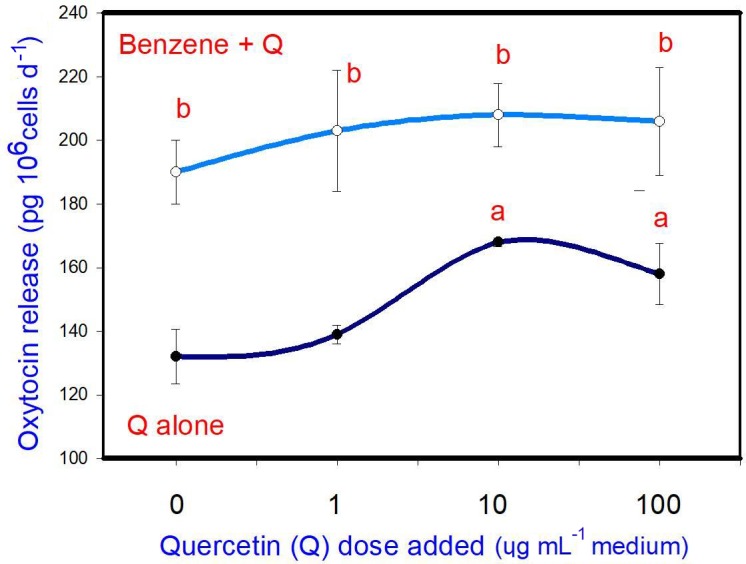
Effects of benzene, quercetin extract, and their combination on the release of oxytocin in cultured porcine ovarian granulosa cells. Parts marked “a” show the effect of quercetin extract (significant difference (P<0.05) between cells cultured without and with quercetin addition at three different levels without benzene), b the effect of benzene (significant difference (P<0.05) between cells cultured with or without benzene for each quercetin dose separately), c the effect of quercetin extract at simultaneous benzene presence (significant difference (P<0.05) between a cell cultured with and without quercetin addition at three different levels with simultaneous benzene addition); effect of benzene alone (20 µg mL${}^{-1}$) is displayed for quercetin dose at 0 µg mL${}^{-1}$.

B (20 µg mL${}^{-1}$) stimulated oxytocin release. Q, when added alone, stimulated oxytocin release (at 10 and 100 µg mL${}^{-1}$, but not at 1 µg mL${}^{-1}$). When administered in conjunction with B, Q did not significantly modify the stimulatory effect of B on oxytocin release at any dose (Fig. 4).

### 5.5 Prostaglandin F

**Figure 5:**
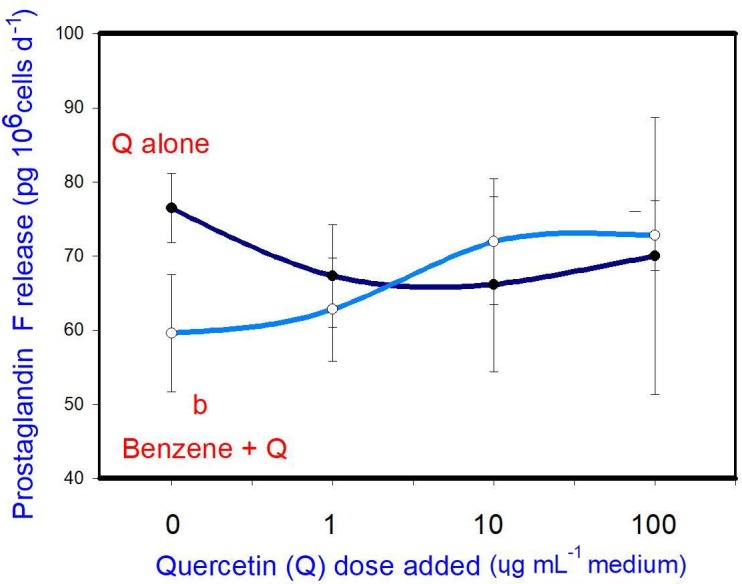
Effects of benzene, quercetin extract, and their combination on the release of prostaglandin F in cultured porcine ovarian granulosa cells. Parts marked “a” show the effect of quercetin extract (significant difference (P<0.05) between cells cultured without and with quercetin addition at three different levels without benzene), b the effect of benzene (significant difference (P<0.05) between cells cultured with or without benzene for each quercetin dose separately), c the effect of quercetin extract at simultaneous benzene presence (significant difference (P<0.05) between a cell cultured with and without quercetin addition at three different levels with simultaneous benzene addition); effect of benzene alone (20 ng ⋅ mL${}^{-1}$) is displayed for quercetin dose at 0 ng ⋅ mL${}^{-1}$

B (20 µg mL${}^{-1}$) inhibited prostaglandin F release. Q when added alone was not able to affect prostaglandin F release at any dose. When administered in conjunction with B, Q did not significantly modify the inhibitory effect of B on prostaglandin F release at any dose (Fig. 5).

## 6 Discussion

### 6.1 Does benzene affect ovarian cell function?

Our results showed that the administration of benzene stimulated proliferation and apoptosis in porcine ovarian granulosa cells. This is the first evidence for direct action of benzene on the ovary. As PCNA is a marker of the S phase of mitosis (Connolly and Bogdanffy, 1993), we expect that benzene may target this phase of the cell cycle. Moreover, the stimulatory action of benzene on cell proliferation observed in our experiments could explain the ability of benzene to induce malignant transformations characterized by increased cell division (Arp et al., 1983). BAX is a known regulator and marker of cytoplasmic apoptosis (Gaumer et al., 2000); therefore, it might be hypothesized that benzene promotes this kind of apoptosis. Furthermore, the ability of benzene to promote both proliferation and apoptosis suggests that benzene can increase ovarian cell turnover. The physiological significance of this effect, as well as the interrelationships between proliferation and apoptosis in ovarian cells when influenced by benzene, requires further clarification. We observed that benzene exerted an inhibitory effect on progesterone and prostaglandin F release and a stimulatory effect on oxytocin release. This is the first demonstration of the effect of benzene on the release of ovarian hormones. These hormones are known regulators of ovarian cell proliferation, apoptosis, steroidogenesis, folliculogenesis, and fecundity (Sirotkin, 2014). Thus, it might be proposed that the previously reported adverse effects of benzene on the female reproductive system (Vara and Kinnunen, 1946; Michon, 1965; Mukhametova and Vozovaya, 1972) may be due to the direct action of benzene on the release of ovarian hormones, which in turn regulate ovarian cell proliferation, apoptosis, and other reproductive processes. Altogether, our study is the first to demonstrate the potential ability for benzene to directly affect ovarian cells by promoting proliferation, apoptosis, and oxytocin release and by inhibiting the release of progesterone and prostaglandin F.

### 6.2 Does quercetin affect ovarian cell functions?

Our observations demonstrated that quercetin inhibits proliferation and apoptosis and also stimulates oxytocin (but not progesterone and prostaglandin) release in porcine ovarian cells.

The studies of Gao et al. (2012) and Ren et al. (2015) observed that quercetin exerted an inhibitory effect on proliferation and a supportive effect on apoptosis in human ovarian cancer cells. Similarly, another study on patients with pancreatic cancer (Angst et al., 2013) suggests a possible benefit of quercetin. However, these results contradict the findings of Leśniak-Walentyn et al. (2013), who demonstrated increased proliferation (Shu et al., 2011) and decreased apoptosis in rabbit (Shu et al., 2011; Tarko et al., 2018) and decreased apoptosis in bovine (Tarko et al., 2018) ovaries cultured with quercetin. The study (Tarko et al., 2018) also demonstrated an inhibitory effect of quercetin on progesterone release. Our results are in partial agreement with all of these studies. The differences in the characteristics of quercetin action on ovarian cell proliferation and apoptosis could be explained by the differences in species (human, rabbits, bovine, porcine), cell health (normal and malignant cells), and markers (PCNA, caspase-3, terminal deoxynucleotidyl transferase, BAX) measured or used in different studies. Oxytocin promotes cell proliferation and steroid hormone release by ovarian cells (Berisha and Schams, 2005; Niswender et al., 2007; Skarzynski et al., 2008; Sirotkin, 2014) and apoptosis in neonatal ovaries (Marzona et al., 2001). During luteolysis, oxytocin as a luteotropic hormone can oppose the luteolytic action of prostaglandin F2 alpha (Gimpl and Fahrenholz, 2001). Our study did not show an association between quercetin and the proliferation and apoptosis or the release of any hormone, which suggests that quercetin probably does not affect proliferation and apoptosis via the release of progesterone, oxytocin, PGF, or PGM. This is the first demonstration of the effect of quercetin on healthy bovine ovaries. Nevertheless, the action of quercetin on ovarian cell proliferation, apoptosis, and oxytocin release that was observed here suggests the involvement and applicability of quercetin in the regulation of porcine and human reproductive processes, including fecundity. It suggests, that the addition of quercetin in animal feed could help improve porcine fertility. However, this hypothesis requires validation with adequate in vivo studies.

### 6.3 Can quercetin modify the effect of benzene on ovarian cell functions?

Some negative effects of environmental stressors on ovarian functions could be prevented or neutralized by certain plants that contain antioxidants and adaptogens (Ungvary et al., 1981; Liang and Yin, 2010). Animal experiments suggest that quercetin, due to its anti-oxidative and anti-apoptotic characters, may provide effective protection against the toxic effects of cadmium (Bu et al., 2011) and dimethyl sulfoxide (Cao et al., 2007). We failed to find any previous research on the potential protective effect of quercetin against the action of benzene or other petrochemical environmental contaminants.

In our experiments, quercetin supported the effect of benzene on progesterone release and did not modify the effect of benzene on proliferation, apoptosis, and oxytocin and prostaglandin F release. Therefore, our observations provide the first evidence that quercetin does not appear to protect ovarian cells against the negative effects of benzene on ovarian cell proliferation (accumulation of PCNA), apoptosis (BAX), and the release of these hormones. Therefore, quercetin cannot be considered protective against benzene action with respect to these processes. Consequently, we may not expect quercetin to protect against benzene, which may induce carcinogenesis by promoting cell division (Arp et al., 1983). Moreover, the cumulative action of quercetin and benzene on ovarian cell progesterone output suggest that quercetin does not protect but rather may even promote the negative effect of this contaminant.

In summary, our observations demonstrate the direct effects of both benzene and quercetin on basic porcine ovarian cell functions (proliferation, apoptosis, and secretory activity). Benzene promoted ovarian cell proliferation, apoptosis, and oxytocin release, and it inhibited progesterone and prostaglandin F release. Quercetin inhibited proliferation and apoptosis, but it stimulated oxytocin. Therefore, quercetin may prove potentially useful in the control of animal and human reproduction, including to enhance fertility or treat reproductive disorders. This is the first demonstration of how quercetin can modify the effect of benzene on ovarian cell function. However, in this paper, quercetin did not prevent but rather promoted benzene action on ovarian function. Therefore, it cannot be used for the prevention of the effects of benzene on these processes. The hypotheses arising from this study require further verification with appropriate in vivo studies.

## Data Availability

The data used in this paper are available on request from the corresponding author (tarko.adam.000@gmail.com).
